# Exploration of barriers to postnatal care service utilization in Debre Libanos District, Ethiopia: A descriptive qualitative study

**DOI:** 10.3389/fgwh.2022.986662

**Published:** 2022-08-26

**Authors:** Kasahun Girma Tareke, Garumma Tolu Feyissa, Yohannes Kebede

**Affiliations:** ^1^Department of Health, Behavior and Society, Institute of Health, Jimma University, Jimma, Ethiopia; ^2^Dornsife School of Public Health, Drexel University, Philadelphia, PA, United States

**Keywords:** postnatal care, barriers, utilization, Debre Libanos, Ethiopia

## Abstract

**Background:**

In Ethiopia, postnatal care (PNC) service utilization was low although many interventions had been implemented. Previous studies showed community-/caregiver-related barriers to PNC service utilization, but limited evidence was available on the health facilities and health care provider-related barriers. Therefore, the study was aimed at exploring both community and health care provider-related barriers to PNC service utilization.

**Methods:**

A descriptive qualitative study was conducted at Debre Libanos District, Ethiopia, from 11 March to 7 April 2019. A purposive sampling technique was used to recruit study participants among recently delivered women (<2 months), health care providers, and community members. A total of five in-depth interviews, 12 key informant interviews, and four FGDs were conducted. Data were audio-recorded, transcribed verbatim, and translated, and inductive thematic analysis was used to analyze the data using the atlas ti.7.1 software.

**Results:**

A total of 51 participants were involved in the study. The findings were organized into two major themes: (1) Community/caregiver-related barriers to PNC service utilization: lack of awareness about PNC, its importance, and schedules; lack of awareness about postnatal danger signs, sociocultural and religious beliefs, topographical and transportation problems, non-functionality of the health developmental armies (HDA); (2) health facility and health care provider-related barriers to PNC service utilization: poor supportive supervision and monitoring, lack of health extension workers' (HEW) commitment, lack of an organized system to notify delivery to HEW, shortage of HEWs, the residence of the HEWs, closure of health posts (HP) on working hours, and non-functionality of HPs.

**Conclusions:**

The study findings underscore the need to develop different strategies and take actions. Therefore, the health centers and district health offices should have to assign the required number of HEWs at HPs, regularly supervise and monitor HEWs, and develop an organized system to facilitate early notification of delivery to HEWs. The HEWs should have to live near the HP, re-organize HDAs, create awareness of maternal and newborn danger signs, and conduct social and behavioral change communications to increase the health-seeking behavior of community members for utilizing PNC services.

## Background

Maternal and newborn mortality had been unacceptably high despite the implementation of many interventions. Globally, there were an estimated number of 295,000 maternal deaths in 2017. The vast majority of these deaths (94%) occurred in low-resource settings. Sub-Saharan Africa and Southern Asia accounted for ~86% (254,000) of the estimated maternal deaths [new, (1)]. On the other hand, there was an estimated number of 2.5 million newborn deaths in 2018 (2). In Ethiopia, maternal and newborn mortality was also high, with a ratio of 412 and 30 maternal (3) and newborn (4) deaths per 1,000 live, respectively.

Most maternal and newborn deaths occurred during the first week of life. This was because existing evidence showed that life-threatening maternal and newborn complications occur during this period. On the other hand, the postnatal period was the most neglected time for the provision of quality services, especially in low- and middle-income countries (5).

Postnatal care (PNC) is a high-impact intervention (4–9) provided for women and newborns to achieve a global plan targeted to reduce maternal and neonatal mortality to <70 and 12 deaths per 100,000 live births, respectively, by 2030 (10). The World Health Organization strongly recommends the provision of PNC service on days 1, 3, and 7, and at 6 weeks to identify and treat postnatal complications, and also to provide the mother with important information on how to care for herself and her child (5).

In Ethiopia, the PNC service is provided at the health facilities, and importantly, the health extension workers (HEWs) provide the service at the health post (HP) and home-to-home to reach all mothers and newborns on days 1, 3, and 7, and at 6 weeks (6–9, 11). On the other hand, there are health developmental armies (HDA) (i.e., organized movement of communities forged through participatory learning and action meetings) that facilitate service delivery and support the HEWs in notifying the presence of delivered women (12) and promoting and developing the health-seeking behavior toward key maternal and newborn health care services (6, 8, 11).

Despite these opportunities, PNC service utilization has been low (4, 13–17). Existing evidence indicates residence, religion, place of delivery, maternal knowledge of danger signs, history of obstetric complications, having antenatal care (ANC) follow-up, birth outcome, educational status, and household wealth as factors affecting PNC service utilization (4, 7, 9, 13–19). However, in the context where the PNC service is provided both at the health facilities and home-to-home, only community-/caregivers-related factors might not be enough to fill the knowledge gaps and needs to address the barriers related to the health facility and health care provider. Therefore, this study aimed to explore barriers related to PNC service utilization from the perspective of the community/caregivers, health facility, and health care providers.

## Methods and materials

### Study setting, period, and approach

A descriptive qualitative study was conducted in Debre Libanos District, North Shoa, Oromia regional state, Ethiopia, from 11 March to 7 April 2019. The district is 90 km far away from Addis Ababa. The population of the district was 45,179, of whom 23,351 and 21,828 were men and women, respectively. Approximately, 19.82% of the population was urban dwellers. Most inhabitants (99.29%) practiced Ethiopian Orthodox Christianity (20). The district had two health centers, eleven HPs, and three private primary clinics. There were four health officers, twenty-two nurses, four laboratory technicians, two druggists, five midwives, and nineteen HEWs (21).

### Study participants and participant recruitment

A purposive sampling technique was used to recruit study participants among women who gave birth within 2 months before data collection (who had or did not have a history of PNC visits), and their families (i.e., husbands, mothers, fathers, mother-in-law, and father-in-law), pregnant women, religious leaders, kebele chairman, HEWs, health workers, and other community members. Participants were recruited based on having perceived rich data on the PNC service. For example, community members, including women who gave birth within 2 months prior to data collection, were recruited based on their role as a newborn caregiver; history of PNC follow-up for the last child; history of perceived maternal and newborn illness; history of newborn death within the postnatal period; history of home delivery; history of seeking care for maternal and newborn health problems during the postnatal period. Family and other community members were also involved in this study based on the social role played within the community in providing care or support to the mother and newborn with close intimacy. In addition, health workers, including health extension workers were included as key informant interviews based on their role in providing PNC service (i.e., at a health facility or through home visits), and monitoring and supervising the service delivery.

### Data collection procedures (instruments, personnel, data collection)

The data were collected using a semi-structured guide. The guide was first developed in the English language and then translated into Afan Oromo and Amharic languages and back-translated into the English language by an independent translator. It contained 8–10 questions that were customized per respondent type. The guiding questions were prepared to start with the general concept and then move to specific issues to cover topics related to (a) community-/caregiver-related barriers; (b) health facility–related barriers; and (d) healthcare provider–related barriers for PNC service utilization.

Data were collected through in-depth interviews, key informant interviews, and focused group discussions (FGD). The in-depth interviews were conducted with five women who gave birth within 2 months before data collection. Additionally, a total of 12 key informant interviews were conducted with HEWs (six), health workers (four), kebele chairman (one), and a religious leader (one). Four FGDs were conducted with a total of forty participants. For each FGD, ~7–12 heterogenous groups of individuals participated. The participants were women who gave birth within 2 years before data collection (who had or did not have a history of PNC visits), and their families (i.e., husbands, mothers, fathers, mother-in-law, and father-in-law), pregnant women, and other community members.

The participants were informed 3 days before data collection. All the interviews and FGDs were conducted in the participant's natural setting. Women or caregivers were interviewed at their homes; kebele chairman, religious leader, health workers, and HEWs were interviewed at their respective offices, and the FGDs were conducted within the community at a place where it was comfortable for all participants.

At the beginning of each interview and group discussion, the purpose of the study and topics of discussions were mentioned to the study participants. Written informed consent was taken from the participants for their willingness to participate. The principal investigator (KGT) was the modulator throughout the data collection. Only the modulator facilitated the in-depth and key informant interviews face-to-face with each participant. However, one research assistant was involved while conducting the FGDs to take a note and record the voices of the participants. On average, the FGDs lasted from 1:15 to 1:41 and the interviews lasted from 21:33 to 43:51 min with the community members, and from 0:39:40 to 1:12 h with health workers and HEWs.

### Data analysis

The inductive thematic analysis was used to analyze the data using the Atlas.ti.7.1 software package, and the codes, categories, and themes were generated from the data. Data analysis commenced during the data collection process. After each data collection, the data were debriefed. Data were transcribed verbatim from audio-recorded material and translated into the English language. Then, reading and re-reading the transcriptions were done to get a whole sense of data. The codebook manual was developed after a line-by-line coding was conducted by the principal investigator and research assistant, separately, starting with transcription having relatively richest data (i.e., data having relatively dense concepts in relation to addressing research questions) and checking the inter-coder consistency. Then, the principal investigator precisely coded the whole data. Potential categories and themes were generated by clustering codes and categories, respectively, to answer the research question. Coding was repeated four times while refining the codebook manual, categories, and themes. Finally, findings were presented with the major themes, categories, and quotations derived from the data.

### Trustworthiness (Rigor)

Different approaches were followed to ensure the trustworthiness of the study findings. First, a guide was pre-tested with three women who gave birth within 2 months and with the three HEWs. Second, the data were debriefed daily together with a research assistant. Third, major thematic areas were raised to the study participants at the end of each data collection for their clarification and verification. Also, the transcriptions and key findings were shared with the HEWs and health workers to check interpretations, and they provided their comments. Fourth, a diversified number of individuals were recruited, and data were collected through three techniques. Fifth, a thick description was provided of the research methodology, interpretation of results, and contributions of a research assistant. Therefore, anyone interested in applying the findings of the study should understand and consider the contextual information provided in the study. Furthermore, the study findings might apply to a setting that is similar to the Ethiopian health care system. Sixth, the consistency and a detailed chronology of overall research activities, processes, and study outputs were audited by colleagues and qualitative research experts. Seventh, *researcher's self-reflectivity and bracketing*: The researchers are public health officers (KGT and GTF) and health education officer (YKL). They have work experience at governmental, private, and non-governmental institutions. They also have experience conducting qualitative and quantitative research. This preconception, knowledge, and skills benefited the researchers to focus on research questions. Additionally, the researchers were not familiar with the study setting, and there was no potential bias that could be introduced if the researchers were from the same location. The subjectivity of the researchers was managed by balancing together with the data, analytic processes, and findings in such a way that the reader can confirm the adequacy of the findings. Additionally, the researchers can speak the local language well, which is used to minimize interpretation bias that could happen from language differences. Eight, the principal investigator spent 1 month in the study setting and created rapport with study participants, and verified the closure of HPs during closed working hours, the residence of HEWs, and lack of regular supervision and monitoring given to the HEWs. Ninth, *negative case analysis*: The contradicting ideas or deviant cases that emerged in the data were analyzed by enquiring in-depth from potential study participants.

### Ethical approval and consent to participate

Ethical approval was obtained from the Jimma University Research Ethical Review Board, Ethiopia with approval Number of THRPG?/301/2019. The right of the study participants was maintained by ensuring non-maleficence and underscoring the benefits of the study. Study participants were informed adequately about the purpose of the study, voluntary participation, and the right to participate or withdraw at any time. To ensure their privacy and autonomy, codes were used in place of their names in connection to the study findings or their responses to discussions or interviews. Enough time was given to them to reflect and provide a detailed explanation of the issue. All of the participants completed the study. Participants' socio-demographic characteristics and written informed consent were taken individually before starting the interview or discussion.

## Results

A total of five in-depth interviews, twelve key informant interviews, and four FGDs were conducted. A total of fifty-two participants were involved in the study.

### Participants' socio-demographics

The demographic characteristics of the participants are summarized in Table 1. The mean age was 37.6 years (range: 21–73 years). The majority (36) of the participants were women. All of them were Ethiopian Orthodox Christianity followers and Oromo in ethnicity.

**Table 1:** Demographic characteristics of participants in Debre Libanos District, North Shoa, Oromia regional state, Ethiopia, 2019.

**Characteristic**	**Category**	***N***	**Characteristic**	**Category**	***N***
Age (years)	20–30	17	Education status	Secondary	4
	31–40	23		Diploma	7
	41–50	7		Degree	1
	>50	5	Religion	Orthodox	52
Gender	Male	15	Ethnicity	Oromo	52
	Female	37	Occupation	Housewife	26
Marital status	Single	5		Merchant	2
	Married	47		HEW	6
Residence	Urban	10		Health worker	4
	Rural	42		Kebele chairman	1
Education status	Illiterate	17		Priest	1
	Primary	17		Farmers	10
	Secondary	6		Government employee	2

### Barriers to PNC service utilization

The study findings were organized into two major themes: community/caregivers, and health facilities and health care providers. The majority of the barriers were mentioned by all groups of participants (i.e., community members, health workers, and HEWs). However, the first two barriers presented in Table 2 were the majority mentioned by participants from the community. A detailed description is found in Table 2.

**Table 2:** Matrix showing barriers to PNC service utilization in Debre Libanos District, North Shoa, Oromia, Ethiopia, 2019.

**Major themes**	**Categories**	**Mentioned by**
Community/caregivers related barriers	Lack of awareness about the PNC, its importance, and schedules	Majorly by community members
	Lack of awareness about the maternal and newborn danger signs	Majorly by community members
	Socio-cultural and religious beliefs	Community members, health workers and HEWs
	Topographical difficulties and transportation problem	Community members and HEWs
	Non-functionality of HDAs	Community members, health workers and HEWs
Health facility and health care provider-related barriers	Lack of regular supervision and monitoring given to HEWs	Community members, health workers and HEWs
	Lack of HEWs commitment	Community members and health workers
	Lack of organized system to notify delivered women to the HEWs	Community members, health workers and HEWs
	Residence of the HEWs, and closure of HPs on working hours	Community members, health workers and HEWs
	Shortage of HEWs	Community members, health workers and HEWs
	Non-functionality of the HPs	Community members and health workers

### Community-/caregiver-related barriers to PNC service utilization

#### Lack of awareness about the PNC, its importance, and schedules

Study participants mentioned that the community members or caregivers had a gap of knowledge or awareness about the PNC, its importance, and schedules. Additionally, it was mentioned that they did not perceive the importance of visiting health facilities or seeking care from health care providers unless the women and newborns faced severe conditions. The caregivers or women also mentioned that the health care providers did not create awareness about these issues, and they appointed the caregivers to visit the health facility on the sixth week to immunize the newborns and get the family planning service. Due to this, it was mentioned that women did not visit the health facility or call the HEWs to their homes to get the service.

“*Visiting the health facility after delivery is not practical in our community due to a lack of awareness of its importance. We learned nothing about the importance of postnatal care and their schedules. We only know that immunization is started on the 45*^*th*^ *day.” (22 years old, female, IDI participant, delivered woman)*

### Lack of awareness of maternal and newborn danger signs

Women, families, and other community members also indicated that they did not know about the maternal and newborn danger signs occurring during the postnatal period. They replied that HEWs or other health workers did not tell about it which made them perceive it as it was not important to have PNC follow-up unless the mother and/or the newborn become severely sick.

“*I do not have any information about the danger signs occurring after deliver on the mother and baby. The health workers did not tell me about these issues during my antenatal follow-up or while we return home after delivery.” (36 years old, female, IDI participant, delivery woman)*

### Sociocultural and religious beliefs

Study participants mentioned two types of sociocultural and religious beliefs that affected PNC service utilization. The first was a religious belief, and among the community members who practiced Ethiopian orthodox Christianity, study participants mentioned that it was forbidden to go out of the home for both the women and newborns before the date of baptism (the 40th day for male newborn and the 80th day for female newborn) due to fear of evil spirit. Therefore, they mentioned that they did not visit the health facilities to seek the PNC service before the date of baptism.

“*After I gave birth, the health extension worker visited us yesterday on the forty-fifth day. This is because, in society's culture, it is forbidden to go out of the home before the date of baptism.” (22 years old, female, IDI participant, delivered woman)*

The second belief mentioned by the study participants was ‘hamechisa' (i.e., a celebration where newborns are taken to and blessed by a traditional healer (i.e., witch) within the first 2 months of life). They mentioned that community members who believe in this culture did not visit the health facilities to seek any health care service before newborns were taken and blessed by the witch. They perceived that unless the newborns were taken and blessed, the witch or traditional healers curse (i.e., a solemn appeal to a supernatural power to inflict harm on someone) the family, especially the delivered mother and newborn, and harmed or died from the evil spirit.

“*At some kebeles, even to take for baptism, there is something called hamu. At those kebeles, newborns do not take vaccines, any treatment, or even not celebrate their date of baptism before going to hamechisa and the witch blesses them.” (34 years old, male, IDI, health worker)*

### Topographical difficulties and transportation problems

Study participants mentioned that there were kebeles that have difficult hills, and no transportation access there to go to the health facilities for getting the service. They responded that in these settings, there was no suitable condition for the HEWs to conduct a home visit, or for the caregivers to go to the health facilities for getting the PNC service. Due to this issue, only community members who lived around or nearby the health post benefited from the service given at the health post, especially where there was only one HEW.

“*Our kebele is very wide, and there is a topographic and transportation problem that makes us unable to take, especially the newborns to the health post. Also, there is only one health extension worker, and because of this case, she only visits delivered mothers who live around the health post, and those who live far from the health post would not gain follow-up service.” (34 years old, male, FGD, community member)*

### Health facility—and health care provider—related barriers to PNC service utilization

#### Supervision and monitoring

Study participants mentioned that there were no regular supportive supervision and monitoring given to the HEWs from the district health office and health centers. Because of this, the HEWs did not open the HPs for service during working hours. As it was mentioned by the study participants, there was a weak health center and HP linkage, and the health workers were not committed to supporting the HEWs. As a result, the community members could not visit the health facilities to get PNC service utilization.

“*One challenge is that the health extension workers do not conduct the activities appropriately at all. There is no monitoring given to them. Other, health posts are not always open for service. Due to these issues, community members do not go to health posts.”(37 years old, female, IDI, health worker)*

#### The HEWs' commitment

Study participants mentioned that the HEWs did not provide PNC service through home-to-home visits or were not available full working hours at the HPs for providing the service. They replied that HEWs were not committed to conducting the activities as expected from them, and they did not provide PNC service routinely but provided it along with the immunization campaigns.

“*If the health post is considered as a health facility, why do the health extension workers live here and provide the service? They [HEWs] are only available here for only two days.” (32 years old, male, FGD, community member)*

“*It is expected for a woman who gave birth to get postnatal care within seven days. However, in our district, almost all of the HEWs travel from here [town] and they only target in providing vaccines for newborns. They provide postnatal service on routine vaccine program or not provide it regularly as needed.” (30 years old, male, IDI, health worker)*

### Residency of HEWs and closure of HPs on working hours

Study participants also mentioned that the HEWs conduct activities traveling from the district town. Because of this case, it was mentioned that they might not go to the HP or reach late, and the HEWs opened the HPs for a maximum of 3 days per week. For example, participants mentioned that the community members/caregivers need to seek care for newborns early in the morning, but HEWs came late. Due to this, they faced challenges in utilizing the service given by the HPs.

“*It is expected that the health extension works should provide PNC service on the first, third, and seventh days, and at six weeks through a home-home visit. However, we do not conduct it as it is expected from them since they are living and work traveling from the district town.” (32 years old, male, IDI, health worker)*

“*The health extension workers live at town and available at the health post three days per week. Even on these days, they came late around 3 or 4 hours [morning] and returned back at eight hours [afternoon]. However, our community members want to take, especially their newborns, to health facilities early in the morning. Therefore, at a time when community members want to return back to home, the health extension workers reach health post.” (37 years old, female, community member)*

### The functionality of health developmental army

Study participants mentioned that the majority of the HDAs were non-functional. It was mentioned that, previously, HDAs had benefited the community in creating awareness, promoting service, and developing behavior of community members toward health care service utilization. However, they mentioned that the HDAs became non-functional, and it was mentioned as a barrier to PNC service utilization.

“*In my perception, the health developmental army has a very essential benefit to get health care service, especially to avoid maternal and newborn death. But it is not available and the supervisors should also have to work together to aware of the community and reestablish it.” (34 years old, male, FGD, community member)*

### Shortage of HEWs

Study participants mentioned that there were two health posts with only one HEW, which made it difficult to reach the delivered mothers and their newborns to provide the PNC service.

“*There are two health posts that have only one health extension worker and one health post with no health extension worker. Therefore, with this condition, it is difficult to utilize the postnatal care service.” (37 years old, female, IDI, health worker)*

### Lack of an organized system for notifying delivered women to HEWs

Study participants mentioned that there was a lack of organized system to notify the presence of delivered women to the HEWs from both the health centers and community members. It was mentioned that the HEWs traced the women from the delivery register every week or every 2 weeks and during immunization campaigns. Due to such cases, study participants mentioned that the caregivers visited the health facilities or HEWs visited them after 6 weeks.

“*We provide PNC when we heard the presence of delivered women. We traced women who gave birth from the delivery register every 15 days or we heard from the community members during immunization campaigns.” (36 years old, female, IDI, HEW)*

“*They (HEWs) did not visit us until our 45*^*th*^ *day but she visited us on this day to provide a vaccine for the baby.” (21 years old, female, IDI, delivered women)*

### Non-functionality of HP

Study participants mentioned that there was one HP with no HEWs and became closed. Due to this, women and newborns did not get PNC service either through a home visit or at the HP.

## Discussion

The study found community/caregiver– and health facility– and health care provider–related barriers to PNC service utilization. The explored barriers were lack of awareness about PNC, its importance, and schedules, lack of awareness of the maternal and newborn danger signs, socio-cultural and religious beliefs, lack of an organized system to notify delivery to HEWs, topographical difficulties and transportation problems, non-functionality of the HDAs, lack of commitment among HEWs, shortage of HEWs, the residence of HEWs and closure of HPs on working hours, poor supportive supervision and monitoring given to HEWs, and non-functionality of HPs.

This study found that there was a lack of awareness about PNC, its importance, and schedules among community members/caregivers to utilize the PNC service. The finding is similar to other study findings conducted in Ethiopia (18, 22–24). Additionally, the study found that women were appointed to visit health facilities on the 45th day to take family planning and immunize the newborn. Therefore, the community members had no awareness of the importance of PNC and its schedules. This implies that the health workers did not consistently follow or adhere to the PNC guidelines and principles. This might indicate that there is a need to provide an update to the health workers, including HEWs through refreshment, in-service, or on-job training about the importance of PNC and its schedules. Furthermore, this underscores the importance of developing strategies and taking actions to create awareness about PNC, its importance, and schedules for community members. This finding is consistent with other study findings reported elsewhere (22, 25).

This study also found that community members, including women, had an awareness gap about maternal and newborn danger signs, as a result, it affected PNC service utilization. This made the community members or caregivers perceive that it was not important to visit the health facilities unless there is a severe maternal and newborn health problem. This might indicate that there is a poor quality of counseling or services provided to the women in the continuum of care starting from pregnancy, during labor and delivery, and postnatal period. Similarly, this underscores that there is a need to provide refreshment, in-service, or on-job training to the health workers, including HEWs on appropriate communication skills and knowledge on the continuum of care (i.e., care provided during pregnancy, labor and delivery, and postnatal period). Moreover, health workers, including HEWs need to be committed to providing quality counseling to pregnant women and family members about danger signs that would appear during these periods. This was similar to study findings gained from different settings (15, 17, 19, 22).

The study found religion and “hamechisa” as barriers to PNC service utilization. Among the Ethiopian Orthodox Christianity followers, it was found that community members did not allow the delivered mothers or newborns out of the home until the date of baptism (40th day for male newborns and 80th day for female newborns). On the other hand, there were community members who believed in a culture called “hamechisa.” Among these community members, women and newborns were first taken within the first 2 months and blessed by the traditional healer. Therefore, in both beliefs, the community members did not seek care from the health facilities or health care providers before Christianization (baptism) and were blessed by the traditional healer, respectively. Due to this problem, they did not utilize the PNC service from the health facilities. This was similar to the study findings conducted in different settings (13, 15, 24). This underscores the importance of conducting social and behavioral change communication to change the perception of community members or caregivers and develop their health-seeking behavior to utilize the PNC service.

This study also found a lack of regular supervision and monitoring given to HEWs as a barrier to PNC service utilization, even if it was expected that they should have to be supervised and monitored every week (26). Because of this, the HEWs were not committed to providing the PNC service. Similarly, lack of commitment among HEW was mentioned as a barrier to PNC service utilization (7, 9). The lack of an organized system that notifies the presence of delivered women timely to HEWs was a barrier to PNC service utilization. The HEWs traced women and newborns from the delivery register every week or every 2 weeks or during the immunization campaigns, which means that PNC was not provided on critical days. However, health care providers and HDAs are expected to notify the presence of delivered women at the health center and home, respectively, to the HEWs to provide the PNC service at home or the HPs (8, 12, 26). Therefore, there is a need to develop a strong system that facilitates the notification of delivered women to HEWs to enhance PNC service utilization. The study also found topographical difficulties and lack of transportation access as barriers to PNC service utilization. Some kebeles had difficulty topographically and no means of modern transportations to go to the health facilities, or suitable to the HEWs to conduct a home visit for providing the service. This was similar to different study findings (7, 18, 24, 27).

The study also found that the non-functionality of HDAs is a barrier to PNC service utilization. As it was mentioned by the study participants, the HDAs provide PNC service, notified delivery to HEWs, identified and referred sick newborns, and carried out social mobilization to increase knowledge, develop the attitude and health-seeking behavior toward maternal and newborn health (8, 11), and support the HEWs in delivering MNCH services (7, 11, 12). However, this study found that the HDAs were not functioning well as what is expected from them. Importantly, appropriate strategies need to be developed and action needs to be taken to re-organize strong HDAs that play a substantial role in enhancing PNC service utilization. Moreover, the provision of close supervision and monitoring is required to make them organized and fully functional.

The shortage of HEWs and the non-functionality of HPs were also explored as barriers to the PNC service provision. There were two health posts with one HEW and one health post with no HEW (i.e., non-functional HP). However, the health extension program acknowledges the availability of at least two HEWs, and all HPs should have to be functional (8, 28). Additionally, the residence of HEWs and closure of HPs during working hours were also identified as barriers to PNC service utilization. Almost all of the HEWs work traveling from the district town, and for this reason, HPs were mostly closed during working hours affecting the service delivery. In contrast, other studies have shown that the majority of HEWs open HPs for five working hours (8, 29). This might happen due to geographical differences or the availability of constructed residences in the previous studies.

## Conclusion

From this study, it was understood that PNC service utilization was affected by the barriers related to community/caregivers, health facility, and health care providers. This underscores the need to develop different strategies and take actions to address them. First, women need to get important knowledge and appropriately seek care during pregnancy, labor and delivery, and postnatal period, given that it is a continuum of care. Second, the community members, including families need to provide social support and encourage her to get adequate knowledge and service during these continuous periods of care. Third, the health centers and district health office should have to assign the required number of HEWs at each HPs, provide regular supportive supervision and monitoring of HEWs, and develop an organized system that facilitates early notification of delivery from both at home and the health facility to the HEWs. On the other hand, the HEWs should have to live in nearby HPs, re-organize the HDAs, create awareness of the maternal and newborn danger signs, conduct social and behavioral change communication to change the perception of the community members or caregivers, and develop their health-seeking behavior to utilize the PNC service.

## Data availability statement

The original contributions presented in the study are included in the article/Supplementary material, further inquiries can be directed to the corresponding authors.

## Ethics statement

The studies involving human participants were reviewed and approved by Jimma University Research Ethical Committee. The patients/participants provided their written informed consent to participate in this study.

## Author contributions

KG: conceptualization, formal analysis, visualization, and writing—original draft. KG, GF, and YK: data curation, methodology, project administration, validation, and writing—review and editing. The authors have confirmed that this is their original work. All authors contributed to the article and approved the submitted version.

## Conflict of interest

The authors declare that the research was conducted in the absence of any commercial or financial relationships that could be construed as a potential conflict of interest.

## Publisher's note

All claims expressed in this article are solely those of the authors and do not necessarily represent those of their affiliated organizations, or those of the publisher, the editors and the reviewers. Any product that may be evaluated in this article, or claim that may be made by its manufacturer, is not guaranteed or endorsed by the publisher.
