# Supplementary material for: Exploration of barriers to postnatal care service utilization in Debre Libanos District, Ethiopia: A descriptive qualitative study
Source: Front Glob Womens Health. 2022 Aug 26;3:986662. doi: 10.3389/fgwh.2022.986662 (PMC9458955; doi:10.3389/fgwh.2022.986662)
Supplement: Supplementary file 1 [file Data_Sheet_1.PDF]

A semi-structured guide

Part I: In-depth interview guide for women who gave birth (English Version)

Name of kebele: ____________________ District: _______________________

Name of data collector: ____

Date of interview: _____ /____/ _____

Time start:__/__:__/___ time end:__/__:____

Place of interveiwee_______________________________

1. Socio-demographic related questions:-
   a. How old are you?
   b. Please would you tell me your educational status?
   c. Please would you tell me your marital status?
   d. Please would you tell me the number of children you have?
   e. Please would you tell me your occupation?
   f. Please would you tell me your profession?
   g. Please would you tell me your ethnicity?
   h. Please would you tell me your religion?
2. Please would you tell me about the your delivery?
3. Please would you tell me about postnatal care?
4. Please would you tell me about your postnatal follow-up?
5. Please would you tell me about postnatal danger signs that might happen on women who gave birth?
6. Please would you tell me about postnatal danger signs that might happen on newborns?
7. Please would you tell me community related barriers that affected the PNC service utilization?
8. Please would you tell me health facility related barriers that affected the PNC service utilization?
9. Please would you tell me health worker related challenges that affected the PNC service utilization?
10. Please would you summarize the key points from our discussion?

Part II: Key informant interview guide for health workers, kebele chairman and religious leaders (English Version)

Name of kebele: ____________________ District: _______________________

Name of data collector: ____

Date of interview: _____ /____/ _____

Time start:__/__:__/___ time end:__/__:____

Place of interveiwee_______________________________

1. Socio-demographic related questions:-
    a. How old are you?
    b. Please would you tell me your educational status?
    c. Please would you tell me your marital status?
    d. Please would you tell me the number of children you have?
    e. Please would you tell me your occupation?
    f. Please would you tell me your profession?
    g. Please would you tell me your ethnicity?
    h. Please would you tell me your religion?
2. Please would you tell me about the utilization of skilled delivery in your catchment?
3. Please would you tell me about the PNC service utilization in your catchment?
4. Please would you tell me community related barriers that affected the PNC service utilization?
5. Please would you tell me health facility related barriers that affected the PNC service utilization?
6. Please would you tell me health worker related challenges that affected the PNC service utilization?
7. Please would you summarize the key points from our discussion?

Part III: FOCUS GROUP GUIDE: For community members (English Version)

1. Please would you tell me about PNC?
2. Please would you tell me your catchment community PNC service utilization?
3. How do you see the provision of PNC in your catchment?
4. Please would you tell me the community/caregivers related barriers that affect the PNC?
5. Please would you tell me health facility related barriers that affected the PNC service utilization?
6. Please would you tell me health worker related challenges that affected the PNC service utilization?
7. Please would you summarize the key points from our discussion?
