# Supplementary material for: Exploration of barriers to postnatal care service utilization in Debre Libanos District, Ethiopia: A descriptive qualitative study
Source: Front Glob Womens Health. 2022 Aug 26;3:986662. doi: 10.3389/fgwh.2022.986662 (PMC9458955; doi:10.3389/fgwh.2022.986662)
Supplement: Supplementary file 2 [file Data_Sheet_2.PDF]

Consolidated criteria for reporting qualitative studies (COREQ): 32-item checklist filled for the manuscript entitled "**Exploration of barriers to postnatal care service utilization in Debre Libanos District, Ethiopia: A Descriptive Qualitative Study**"

No. Item	Guide questions/description	Reported on Page #
**Domain 1: Research team and reflexivity**		
Personal Characteristics		
1. Interviewer/facilitator	Which author/s conducted the interview or focused group?	Kasahun Girma Tareke: reported as principal investigator on method, page 5, line 9.
2. Credentials	What were the researcher's credentials? E.g. PhD, MD	**Kasahun Girma Tareke** MPH in health promotion and human behavior; Lecturer **Garumma Tolu Feyissa** MPH in health education and promotion; PhD in Evidence based health care; Associate Professor.. **Yohannes Kebede Lemu** MPH in health education and promotion; now PhD student in health promotion and communication; Associate Professor. Author's information, page 6.
3. Occupation	What was their occupation at a time of study?	KGT: student, data collector; YKL: PhD student, Lecturer and supervisor; GTF: Distance education director, Lecturer and supervisor

4. Gender	Was the researcher male or female?	Male
5. Experience and training	What experience or training did the researchers have?	The researchers have experience in conducting, providing technical assistance and consultancy in areas of qualitative research. They also took special training and are trainers in the areas of qualitative research, including systematic review trainings. Some of these were reported on method, page 6, line 16-18.
Relationship with participants		
6. Relationship established	Was a relationship established prior to study commencement?	No
7. Participant knowledge about the interviewer	What did the participants know about the researcher? E.g. personal goals, reasons for doing the research	Participants were aware about the goal, purpose and objective of the study and detail information was provided on the participant information sheet and Consent Form. Please found on page 7.
8. Interviewer characteristics	What characteristics were reported about the interviewer/facilitator? e.g. Bias, assumptions, reasons and interests in the research topic	It was clearly mentioned how the principal inveestigator and other authors managed any bias or assumptions during the interview or FGDs or analyzing and interpreting the data. Reprted on method, page 7.

**Domain 2: study design**		
Theoretical framework		
9. Methodological orientation and Theory	What methodological orientation was stated to underpin the study? e.g. grounded theory, discourse analysis, ethnography, phenomenology, content analysis	In the method section of the manuscript, it was stated the study approach was framed based on a descriptive qualitative study design. Method, page 4.
Participant selection		
10. Sampling	How were the participants selected? e.g. purposive, convenience, consecutive, snowball	Participants were recruited using purposive sampling technique; Method, , page 4.
11. Method of approach	How were participants approached? e.g. face-to-face, telephone, mail, email	Face to face: Methods, page 5.
12. Sample size	How many participants were in the study?	Fifty two: Results, page 5.
13. Non-participation	How many people refused to participate or dropped out? Reasons?	All of the participants completed the study.
Setting		
14. Setting of data collection	Where was the data collected? e.g. home, clinic, workplace	At participants natural setting.<br>For example, IDI with delivered mother and religious leader at their respective home; IDI with health worker and kebele chairman at their respective office; FGD at kebele level [within the community]: method section, page 5.
15. Presence of non-participants	Was anyone else present besides the participants and researchers?	No

16. Description of sample	What are the important characteristics of the sample? e.g. demographic data, date	Detail important characteristic description of the sample was given: Result, page 8.
Data collection		
17. Interview guide	Were questions, prompts, guides provided by the authors? Was it pilot tested?	The guides were developed by the authors :Method, page 4.
18. Repeat interviews	Were repeats interviews carried out? If yes, how many?	No
19. Audio/visual recording	Did the research use audio or visual recording to collect the data?	Yes. It was audio-recorded: Method page 5.
20. Field notes	Were field notes made during and/or after the interview or focus group?	Yes. Method, page 5.
21. Duration	What was the duration of the interviews or focus group?	The FGDs lasted from 1:15 to 1:41 hour and the interviews with community members from 21:33 to 43:51 minutes; with health workers from 0:39:40 to 1:12 hour: Method, page 5.
22. Data saturation	Was data saturation discussed?	Yes. Method, page 5.
23. Transcripts returned	Were transcripts returned to participants for comment and/or corrections	Yes: it was shared to key informants and comments also provided on it: Method, page 6, line 5-8.
**Domain 3: analysis and findings**		
Data analysis		
24. Number of data coders	How many data coders coded the data?	Two: methods, page 5, line 24-25.

25. Description of the coding tree	Did authors provide a description of the coding tree?	No.
26. Derivation of themes	Were themes identified in advance or derived from the data?	Since this is a descriptive qualitative approach, the themes were derived from the data; not identified in advance: Method, page 5, line 28 and 29.
27. Software	What software, if applicable, was used to manage the data?	Atlas ti.7.1 software package was used to manage the data: Method, page 5, line 22.
28. Participant checking	Did participants provide feedback on the findings?	Yes: Method, page 5, line 5-8.
Reporting		
29. Quotations presented	Were participant quotations presented to illustrate the themes/findings? Was each quotation identified? e.g. participant number	Yes: Result, page 10-14.
30. Data and findings consistent	Was there consistency between the data presented and the findings?	Yes: Result section and discussion section , page 8-14.
31. Clarity of major themes	Were major themes clearly presented in the findings?	Yes: The summary of major themes with their categories was presented in the results section, page 8-14.
32. Clarity of minor themes	Is there a description of diverse cases or discussion of minor themes?	Yes: Result, page 7-14.
